# A Rare Cause of Unilateral Central Retinal Vein Occlusion in a Young Patient: Type III Mixed Cryoglobulinemia

**DOI:** 10.1155/2016/1949362

**Published:** 2016-06-23

**Authors:** Sibel Doguizi, Mehmet Ali Sekeroglu, Mustafa Alpaslan Anayol, Pelin Yilmazbas

**Affiliations:** Ulucanlar Eye Training and Research Hospital, Department of Ophthalmology, 06340 Ankara, Turkey

## Abstract

*Purpose*. To report a young male with unilateral central retinal vein occlusion (CRVO) associated with cryoglobulinemia.* Case Presentation*. A 33-year-old male without any known systemic or ocular disorder was admitted to our clinic with a complaint of visual loss for three days in his left eye. Based on the clinical, laboratory, and ophthalmological assessments, we diagnosed this case as type III mixed cryoglobulinemia with unilateral CRVO with macular edema. For treatment, two intravitreal ranibizumab injections were administered monthly and oral prednisone (64 mg/day) was begun. Subsequently, cryoglobulins became undetectable, macular edema decreased, and the visual acuity improved to 20/32 over an 8-week period. At 24 weeks, the patient's visual acuity remained 20/32 and no recurrence was observed while the patient was still on prednisone (16 mg/day).* Conclusion*. Cryoglobulinemia should be considered in the differential diagnosis of the patients with CRVO.

## 1. Introduction

Central retinal vein occlusion (CRVO) is a major cause of significant visual loss and is a disease predominantly of the elderly, with major risk factors being age, hypertension, hyperlipidemia, and diabetes mellitus. CRVO is uncommon in younger adults and includes unusual causes such as hypercoagulable conditions, hyperviscosity syndromes, and infectious and noninfectious vasculitic diseases [1].

Cryoglobulinemia is characterized by the presence of serum immunoglobulins that precipitate with cold temperature and resolubilize with warming [2]. Cryoglobulins are classified into three types: type I cryoglobulinemia involves monoclonal immunoglobulin, usually immunoglobulin M (IgM) or, less frequently, immunoglobulin G (IgG) or light chains; types II and III cryoglobulinemia (mixed cryoglobulinemia) are immune complexes composed of monoclonal (type II) or polyclonal (type III) immunoglobulin (IgM) with rheumatoid factor (RF) activity [3]. Reported chorioretinal manifestations of cryoglobulinemia include central serous chorioretinopathy [4], retinal vein occlusion [5, 6], and Purtscher-like retinopathy [7]. In this report we describe a young male with unilateral CRVO associated with cryoglobulinemia.

## 2. Case Report

**Figure 1:**
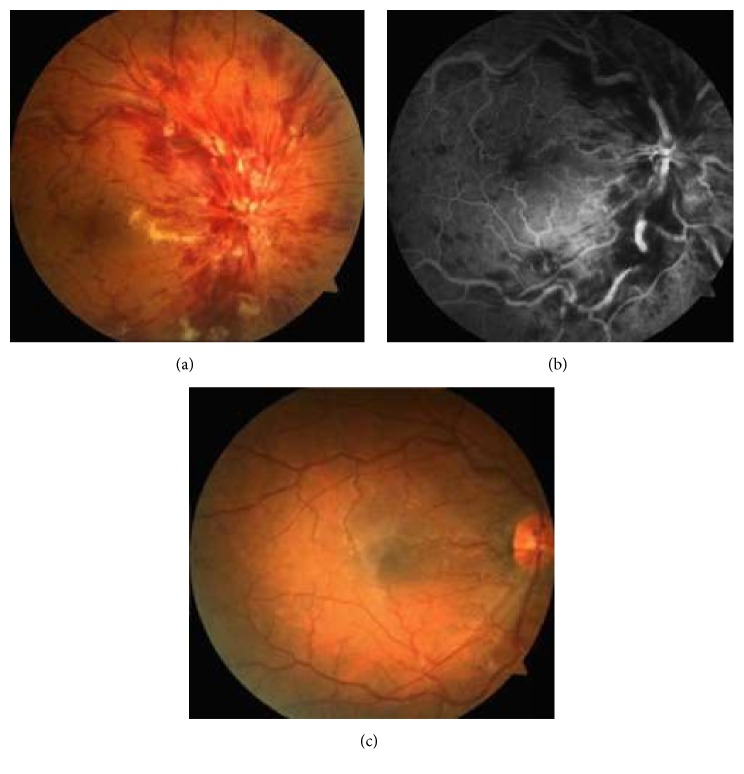
(a) Fundus photograph of the left eye shows central retinal vein occlusion with disk edema, dilated retinal veins, peripapillary cotton-wool spots, and hemorrhages. (b) Fluorescein angiogram (1 minute and 14 seconds after injection of the dye) photograph of the left eye shows central retinal vein occlusion with marked delay in arteriovenous transit time, masked by retinal hemorrhages, vessel wall staining, and a few small patches of retinal capillary obliteration. (c) Fundus photography of the left eye after 8 weeks shows that superficial cotton-wool spots and retinal hemorrhages were all resolved.

**Figure 2:**
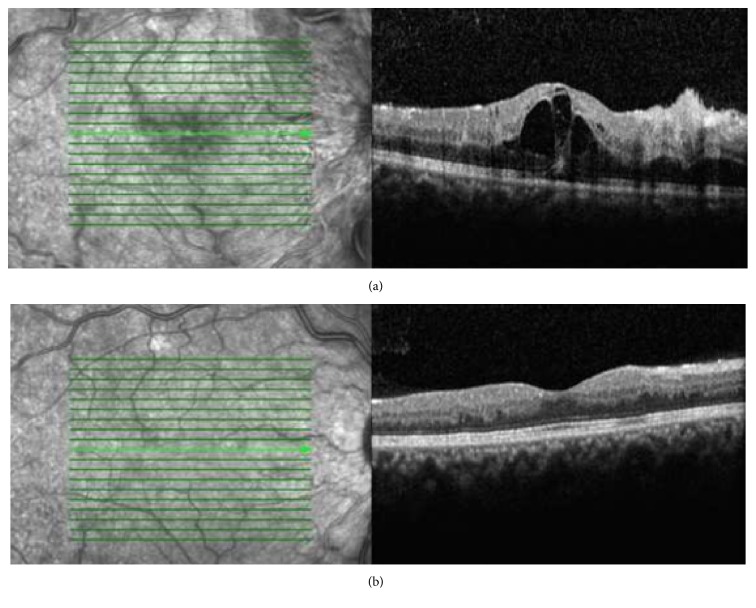
(a) Optical coherence tomography of the left eye shows macular edema (central foveal thickness: 437 *μ*m). (b) Optical coherence tomography of the left eye after 8 weeks (central foveal thickness: 242 *μ*m) (after two monthly ranibizumab injections).

A 33-year-old male without any known systemic or ocular disorder was admitted to our clinic with a complaint of visual loss for three days in his left eye. Visual acuity was 20/20 in the right eye and 20/250 in the left eye. Intraocular pressure was 15 mmHg bilaterally. Anterior segment and the slit-lamp examination were unremarkable in both eyes. Funduscopic examination revealed severe optic disk edema, superficial retinal hemorrhages with cotton-wool spots in all quadrants, and markedly engorged retinal veins in the left eye (Figure 1(a)). Fluorescein angiography (1 minute and 14 seconds after injection of the dye) showed CRVO with marked delay in arteriovenous transit time, masked by retinal hemorrhages, vessel wall staining, and a few small patches of retinal capillary obliteration (in areas of cotton-wool spots) (Figure 1(b)). Optical coherence tomography of the left eye showed macular edema (central foveal thickness: 437 *μ*m) (Figure 2(a)). Further investigation revealed no risk factors associated with central retinal vein occlusion, such as diabetes, hypertension, hypercholesterolemia, or increased body mass index. Hypercoagulable conditions were investigated and no pathological findings were detected in hyperhomocysteinemia, protein S deficiency, protein C deficiency, factor V Leiden mutation, methylenetetrahydrofolate reductase gene mutation, and antithrombin levels. Causes of vasculitis (infectious and noninfectious) were also investigated. The erythrocyte sedimentation rate was 10 mm/h and a chest X-ray was normal. Syphilis, Lyme disease serological markers, and the interferon-*γ* release assay (QuantiFERON®-B Gold-in-Tube; Cellestis, Santa Clarita, CA, USA) were negative. Screening for anti-neutrophil cytoplasmic antibodies and anti-cardiolipin antibodies was negative. Serum is tested for cryoprecipitation by daily inspection at 4°C for 7 days and type III serum cryoglobulins (0.9 mg/mL) were detected. Quantitation of total serum protein and immunoglobulins, complement levels, and RF activity were also performed. RF activity and type III mixed cryoglobulinemia contained polyclonal immunoglobulins (IgM) were detected. RF level was high (2212 IU; normal < 20 IU) and C3 complement level was low (0.26 g/L; normal 0.71–1.56 g/L). Hepatitis C virus and hepatitis B virus serology were negative. After detailed systemic examination, systemic finding of leukocytoclastic cutaneous vasculitis, manifesting as palpable purpura (main cutaneous manifestation of mixed cryoglobulinemia) on the legs, was detected. We learned from the patient that he had started work in a cold storage facility 1 month prior to his visit. No other complication of cryoglobulinemia, underlying rheumatic disease, or chronic infection was found. We diagnosed this case as type III mixed cryoglobulinemia with unilateral CRVO with macular edema. Two intravitreal ranibizumab injections were administered monthly in 2 months. After rheumatology consultation, oral prednisone (64 mg/day) was begun and avoidance of cold was recommended. Subsequently, cryoglobulins became undetectable, the patient's visual acuity improved to 20/32, and superficial cotton-wool spots and retinal hemorrhages all resolved over an 8-week period in the left eye (Figure 1(c)). Macular edema also decreased (central foveal thickness: 242 *μ*m) (Figure 2(b)). At 24 weeks, the patient's visual acuity remained 20/32 and no recurrence was observed while the patient was still on prednisone (16 mg/day).

## 3. Discussion

Type III (mixed) cryoglobulinemia, composed of RF activity and different immunoglobulins, can occur in patients with rheumatic diseases, with chronic infections, and especially with persistent hepatitis most commonly caused by infection with hepatitis C. Cryoglobulinemia is said to be essential when there is no identifiable underlying disease. In our case, we did not find any associated rheumatic diseases or infections, and we considered the case as essential cryoglobulinemia.

CRVO is a well-known complication of paraproteinemias and other hyperviscosity states. We found only two reports of retinal vein occlusion associated with cryoglobulinemia: one involving bilateral central retinal vein occlusion [5] and the other involving branch retinal vein occlusion with central serous chorioretinopathy [6]. This is the first case report of unilateral retinal vein occlusion associated with type III (mixed) cryoglobulinemia.

Cryoglobulins are abnormal antibodies, which precipitate from the serum at low temperatures and act as immune complexes that deposit on the endothelium of small and medium size blood vessels to cause vasculitis. This was probably the mechanism of CRVO in our case. We applied two monthly intravitreal ranibizumab injections and, after rheumatology consultation, oral prednisone (64 mg/day) was also begun. Macular edema, superficial cotton-wool spots, and retinal hemorrhages all resolved over an 8-week period in the left eye. Steroids were slowly decreased and maintained at 16 mg/day without recurrence during a 24-week follow-up.

Clinicians should, therefore, consider cryoglobulinemia as a rare potential association with central retinal vein occlusion.
